# The Resistance Responses of Potato Plants to Potato Virus Y Are Associated with an Increased Cellular Methionine Content and an Altered SAM:SAH Methylation Index

**DOI:** 10.3390/v13060955

**Published:** 2021-05-21

**Authors:** Nadezhda Spechenkova, Igor A. Fesenko, Anna Mamaeva, Tatyana P. Suprunova, Natalia O. Kalinina, Andrew J. Love, Michael Taliansky

**Affiliations:** 1 Shemyakin-Ovchinnikov Institute of Bioorganic Chemistry of the Russian Academy of Sciences, 117997 Moscow, Russia; rysalka47@gmail.com (N.S.); fesigor@gmail.com (I.A.F.); annettest@yandex.ru (A.M.); kalinina@belozersky.msu.ru (N.O.K.); 2 Doka-Gene Technologies Ltd., Rogachevo, 141880 Moscow, Russia; suprunova@gmail.com; 3 Belozersky Institute of Physico-Chemical Biology, Lomonosov Moscow State University, 119991 Moscow, Russia; 4 The James Hutton Institute, Invergowrie, Dundee DD2 5DA, UK; andrew.love@hutton.ac.uk

**Keywords:** potato virus Y, isobaric tags for relative and absolute quantitation (iTRAQ), methionine cycle, plant virus resistance

## Abstract

Plant-virus interactions are frequently influenced by elevated temperature, which often increases susceptibility to a virus, a scenario described for potato cultivar Chicago infected with potato virus Y (PVY). In contrast, other potato cultivars such as Gala may have similar resistances to PVY at both normal (22 °C) and high (28 °C) temperatures. To elucidate the mechanisms of temperature-independent antivirus resistance in potato, we analysed responses of Gala plants to PVY at different temperatures using proteomic, transcriptional and metabolic approaches. Here we show that in Gala, PVY infection generally upregulates the accumulation of major enzymes associated with the methionine cycle (MTC) independently of temperature, but that temperature (22 °C or 28 °C) may finely regulate what classes accumulate. The different sets of MTC-related enzymes that are up-regulated at 22 °C or 28 °C likely account for the significantly increased accumulation of S-adenosyl methionine (SAM), a key component of MTC which acts as a universal methyl donor in methylation reactions. In contrast to this, we found that in cultivar Chicago, SAM levels were significantly reduced which correlated with the enhanced susceptibility to PVY at high temperature. Collectively, these data suggest that MTC and its major transmethylation function determines resistance or susceptibility to PVY.

## 1. Introduction

Plant viruses are responsible for a broad range of diseases which cause severe crop losses and are thus a serious threat to sustainable agriculture. The increasingly frequent emergence and re-emergence of viral diseases is often associated with the high capacity of viruses to rapidly evolve, a situation worsened by global climate change, which can influence vector transmission and plant susceptibility to infection [1]. Being small obligate intracellular parasites, viruses typically recruit or subvert the functions of the host plant cell to facilitate virus replication within the infected cell, prior to their systemic spread throughout the host plant; such molecular plant-virus interplays determine whether virus infection is successful or not. When the virus can successfully infect host plants, this is known as a compatible interaction [2]. In contrast with some host/virus combinations, the interaction may be incompatible, which is typified by the triggering of antiviral immune mechanisms that limit virus replication and spread (this is often observed with R (resistance) gene-mediated virus-host interplays) [2]. While there is an orchestrated cross-talk of virus components with host plant machinery during such interactions, there can be an associated dynamic remodelling of host metabolism during the infection process [3]. A major metabolic process affected by plant virus infections is the methionine cycle (MTC) [4,5].

With regards to host-virus interactions, the MTC is closely associated with RNAi, which is a versatile, sequence-specific mechanism for regulating endogenous gene expression and can degrade foreign (viral) RNAs. RNAi-based defence responses involve the processing of virus-derived double-stranded RNA into small interfering RNAs (siRNAs), which can direct plant enzyme complexes to target the decomposition of homologous viral RNAs [6,7,8,9,10]. Specifically, siRNAs, as pivotal components of RNAi, are stabilized by the MTC-dependent methyltransferase Hua enhancer 1 (HEN1), which methylates them using SAM as methyl donor [11]. Interplay between the MTC and HEN1-mediated activity in RNA silencing may be modulated by some viruses such as potyviruses. Antiviral RNAi-based defence against potyviruses is suppressed by the potyviral helper component-proteinase (HC-Pro) [12]. HC-Pro has also been shown to directly interact with SAMS and SAHH, two major MTC enzymes [12,13], which presumably leads to disruption of the MTC, and SAM production, in particular. This therefore suggests that one of the mechanisms by which HC-Pro exerts its RNAi suppression activities is inhibition of siRNA methylation by HEN1 due to the lack of SAM [4,12].

The MTC is also related to some other metabolic processes implicated in virus–plant interactions. For example, conversion of HCY into MET is a key check point between two distinct pathways either in the direction of MET biosynthesis or toward biosynthesis of polyamines or ethylene [4]. Ethylene is a plant hormone which, as with some other hormones, plays important roles in plant responses to virus infections (reviewed in [14]).

Potato virus Y (PVY), the type species of the genus *Potyvirus*, is one of the most economically important pathogens of potato, and it occurs worldwide [15]. Interestingly host-PVY interactions, as with many other host-virus interactions, are temperature-sensitive and may be regulated by elevated temperature in a cultivar-specific manner, possibly via the modulation of host defence responses [5,16]. For example, elevated temperatures significantly increase the susceptibility of potato cv. Chicago to PVY compared to normal temperature, resulting in a greater degree of PVY infection.

Using proteomic transcriptional and metabolomic analyses, we have recently shown that the main enzymes of MTC are down-regulated in PVY-infected Chicago plants at higher temperatures, causing consistent and concerted reduction in the levels of the major MTC metabolites including the methyl group donor SAM. This suggests that the enhanced susceptibility of Chicago potato plants to PVY at high temperatures may at least be partially triggered by the MTC perturbation and concomitant reduction in transmethylation activities [5].

In contrast to cv. Chicago, potato cv. Gala plants display resistance to PVY at both normal and rising temperatures [16], suggesting that the effect of temperature on virus resistance in different cultivars may be manifested differentially. The major goal of this work was therefore to elucidate mechanisms of cultivar-specific antiviral resistance in potato and its dependence on temperature. More specifically, we aimed to explore the role of the MTC in plant virus resistance by analysing the responses of Gala plants to PVY infection at different temperatures using comparative proteomic, transcriptional and metabolic approaches.

## 2. Materials and Methods

The methods used in this work were essentially similar to those described in detail in our previous paper [5].

### 2.1. Virus, Plants, and Growth Conditions

PVY (ordinary strain, PVY^O^) was propagated in *Nicotiana tabacum* according to [17]. Four-week-old potato plants (*Solanum tuberosum* L.; cv. Gala) were inoculated with PVY and left to grow in a controlled environment chamber (Pol-Eko-Aparatura, Wodzisław Śląski, Poland) which was set at a photoperiod of 16/8 h day/night at a relative humidity of 60% with a light fluence of 250 µmol m^−2^ s^−1^. At two dpi (days post-inoculation), half of the plants were transferred to 28 °C, while the other half remained at 22 °C. Tissue samples were harvested at different time points (8 and 14 dpi for proteomic analysis; 5, 8 and 14 dpi for gene expression analysis; 8, 10 and 14 dpi for metabolite measurements) from three non-infected mock-inoculated plants or from the systemically infected leaves of three virus-inoculated plants. From each plant, two leaves were collected and pooled together; this sampling method was used in four independent experiments and the harvested material was processed and analysed according to the protocols described below.

### 2.2. Protein Extraction, Trypsin Digestion and Isobaric Tag for Relative and Absolute Quantitation (Itraq) Analysis

Proteins were isolated using phenol extraction [18], quantified via Bradford protein assays (Bio-Rad, Hercules, CA USA) and reduced in 5 mM DTT for 30 min at 50 °C and then alkylated by incubating with iodoacetamide. The proteins were then digested by incubating with trypsin (Promega, Madison, WI, USA), and the resultant peptides were labelled according to the 8-plex iTRAQ kit manufacturer’s recommendations (ABsciex Inc., Foster City, CA, USA). The samples derived from material collected at 8 dpi were labelled as follows: iTRAQ reagents 115, 116, 117 and 118, 119, 121 were used to label peptides from mock-inoculated plants incubated at 22 °C (Mock22); peptides from virus-infected plants grown at 22 °C (PVY22) were labelled with iTRAQ reagents 113, 114, 117, and those from virus-infected plants grown at 28 °C (PVY28) were labelled with reagents 118, 119, 121. Peptides from mock-inoculated plant samples collected at 14 dpi were labelled using iTRAQ reagents 113, 114, 115. The corresponding PVY22 and PVY28 samples were labelled with 116, 117 and 121 isobaric tags. The tagged peptides were combined as follows: Mock22 (118, 119, 121) with PVY22 (113, 114, 117) and Mock22 (115, 116, 117) with PVY28 (118, 119, 121) for 8 dpi samples; Mock22 (113, 114, 115) with PVY22 (116, 117, 121) or PVY28 (116, 117, 121) for 14 dpi samples.

### 2.3. LC-MS/MS Analysis and Protein Identification and Quantification

The mass-spectrometry analysis was performed as described in Fesenko et al., 2021 [5]. Reverse-phase chromatography was performed with an Ultimate 3000 Nano LC System (Thermo Fisher Scientific, Waltham, MA, USA), which was coupled to a Q Exactive HF benchtop Orbitrap mass spectrometer (Thermo Fisher Scientific, Waltham, MA, USA). Peptides were separated using an Acclaim PepMap 100 C18 (75 μm × 50-cm) column (Thermo Fisher Scientific, Waltham, MA, USA).

Tandem mass spectra were analysed by PEAKS Studio version 8.0 software (Bioinformatics Solutions Inc., Waterloo, Canada). The custom database was built from Phytozome database *S. tuberosum* combined with chloroplast and mitochondrial proteins (39,809 records). The database search was performed with the following parameters: a fragmentation mass tolerance of 0.05 Da; parent ion tolerance of 10 ppm; fixed modification—carbamidomethylation; variable modifications—oxidation (M) and acetylation (Protein N-term). The resulting protein list was filtered by a 1% false discovery rate (FDR). PEAKS Q was used for iTRAQ quantification. Normalization was performed by averaging the abundance of all peptides. Median values were used for averaging. Given that iTRAQ quantification typically underestimates the degree of real fold changes between two samples [19], differential protein screening (determined by the ratio in the PVY-infected samples and untreated control (mock-inoculated plants at 22 °C) was performed using a fold change ratio >1.2 (for upregulated DEPs) or <0.83 (for downregulated DEPs) and *p* < 0.01 (one-way ANOVA).

### 2.4. Bioinformatic Analysis

Protein–protein interaction networks were constructed using STRING v10 [20,21]. The strength of the protein interactions was set at the default option of 0.4. Cytoscape software [22] was used to visualize the protein interaction networks, and the functions and pathway enrichment of candidate DEGs were analyzed using g:Profiler [23,24].

### 2.5. RNA Extraction and Real Time Quantitative RT-PCR (RT-qPCR)

Species-specific prefixes (St) are used in this section and figure legends to define mRNAs corresponding to the *S. tuberosum* genes: *StMS, StSAHH, StSAMS, StCBL, StSHM, StSAMDM, StEF-1α* and *StCox.* However, for simplicity, in the main text of the manuscript, this “St” nomenclature is not used for genes, mRNAs or proteins. Total RNA was isolated as described previously [16]. DNase-treated RNA was reverse transcribed into cDNA using the SuperScriptTM First-Strand Synthesis System for RT-PCR (Invitrogen), with either an oligo-dT primer (for host plant-specific mRNAs) or a PVY-specific primer (see Table S1). The primer pairs for SYBR green-based real-time PCR analysis of PVY RNA and host mRNAs (which are listed in Table S1) were designed using both Plant Genomics Resource Phytozome 12 [25] and PRIMER EXPRESS software (ThermoFisher Scientific, Waltham, MA, USA). The Ct values for PVY RNA and each mRNA of interest were normalized using two internal reference genes encoding StEF-1α [26] and cytochrome c oxidase subunit 1 (StCOX) [27]; primers are listed in Table S1. The average Ct values of the two reference genes was used to analyse PVY and host mRNA levels.

### 2.6. Analysis of MTC-Related Metabolites

To detect and quantify the contents of SAH, SAM, HCY and MET, approximately 50 mg of fresh tissue from the leaves of three mock- and infected virus-inoculated plants were collected, pulled together and ground in phosphate buffered saline (0.01 M, pH 7.2). The samples were centrifuged at 12,000× *g* for 15 min, and the SAM, SAH and HCY content of the supernatant of the extracts was measured by ELISA according to the recommendations of the Homocysteine (HCY) ELISA kit and S-Adenosylmethionine (SAM) and the S-Adenosylhomocysteine (SAH) ELISA combo kit manufacturers (Cell Biolabs, Inc., CA, USA). The amount of MET was quantified using a Methionine (Fluorometric) assay kit (Abcam, Cambridge, UK) according to the manufacturer’s instructions.

### 2.7. Statistics

Statistical analysis was performed on four biological replicates. Statistical analyses and bar plots were made using Python version 3.7.5 [28]. For two- or multiple-way ANOVA, Tukey honestly significant difference (HSD) tests based on multiple comparisons of means were deployed to determine the pairwise comparisons which were statistically significant. Differences were considered to be significant if the *p* value was <0.05.

## 3. Results

### 3.1. Protein Profiles of PVY-Infected Gala Plants at Normal and High Temperature

In previous work, we investigated the effect of moderately high temperature (28 °C) on the development of PVY infection in relatively thermotolerant Gala plants [16]. This temperature was chosen to mimic the mild heat stress that may arise due to global warming. We showed that Gala displays a distinct type of resistance to PVY, whereby the virus, in spite of its ability to infect plants systemically, does not develop any apparent symptoms and replicates to very low levels [16]. Moreover, in contrast to Chicago plants [16], the resistance in Gala is not affected by elevated (28 °C) temperature.

To explore the underlying mechanisms responsible for resistance to PVY in Gala plants (which is thought to be temperature-independent), we used iTRAQ-based quantitative comparative proteomic technology. iTRAQ analysis was conducted at an early time point (8 dpi), when PVY starts to invade plants systemically, and at a 14 dpi time point, which represents a later stage of infection when metabolic changes may be highly pronounced. A total of 22,833 unique tryptic peptides were identified (Table S2), which were assigned to 5939 protein groups from a custom database. The numbers of peptides and corresponding protein groups identified by LC-MS/MS analysis for both control mock-inoculated and plants infected with PVY at 22 °C or 28 °C are indicated in Table 1. To identify differentially expressed (DE) proteins (FC > 1.2, *p* < 0.01 ANOVA) we performed differential protein screening determined by the ratio of the protein expression levels in the PVY-infected samples to those in corresponding untreated controls (mock-inoculated plants at 22 °C at either 8 or 14 dpi, respectively) as described in Materials and Methods.

In PVY-infected plants grown at normal temperature (22 °C), we identified 43 DE protein groups including 11 up-regulated and 32 down-regulated proteins at 8 dpi, and 189 protein groups (106 up-regulated and 83 down-regulated) at 14 dpi (Table S3).

At 8 dpi, among the population of up-regulated DEPs, several chloroplast proteins including photosystem I P700 apoprotein A1, photosystem II proteins D1 and D2, chlorophyll a-b binding protein 6A, photosystem II 22 kDa protein, were detected (Table S3). It is known that chloroplasts and some of their proteins facilitate defence responses against various plant viruses [29]. This suggests that the increase in quantities of chloroplast proteins in response to PVY, may be a factor determining the resistance of Gala to PVY. Other proteins that were up-regulated due to these conditions were WRKY transcription factor an Armadillo/beta-catenin repeat family protein, which are involved in plant stress responses [30] and SAM (S-adenosylmethionine)-dependent methyltransferase (SAMDM) (Table S3) which belongs to a large group of enzymes that catalyse a variety of methylation reactions via the transfer of methyl groups from SAM on various proteins and nucleic acids. As with enzymes of the MTC, SAMDM may play important role in plant antivirus defence [4,5].

Among the proteins down-regulated at normal temperature (22 °C) in PVY-infected Gala plants at 8 dpi, we identified ribosomal proteins, glutathione S-transferase/peroxidase and profilin. Glutathione S-transferase/peroxidase is involved in antioxidation processes in the plant cell. Its deficiency at 8 dpi may permit the formation of reactive oxygen species (ROS), which could have antiviral activity [31] and therefore facilitate viral replication. Proflin is an actin-binding protein involved in the dynamic turnover and reconstruction of the actin cytoskeleton [32] and as such may be required for efficient intra- and intercellular trafficking of the virus [33]. Thus, down-regulation of both these proteins may disturb some virus functions and hence enforce PVY resistance.

**Figure 1:**
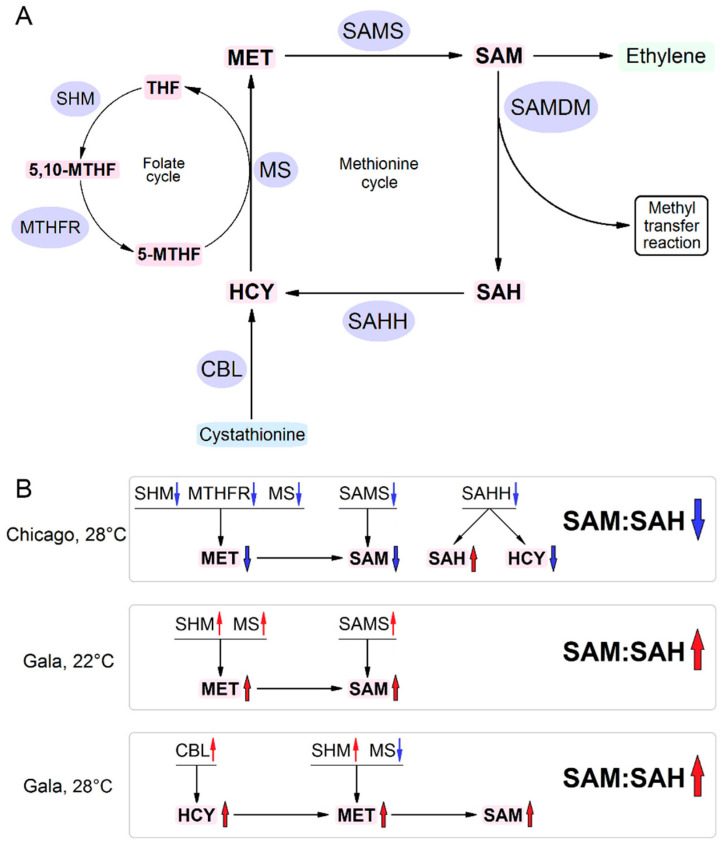
A tentative model showing possible mechanisms determining susceptibility or resistance of potato plants to PVY. (**A**) Schematic representation of the methionine cycle (MTC) in healthy cells. Metabolite (shown in pink) flow directions are presented by arrows, while the enzymes involved in their bioconversion are marked in blue. CBL, cystathionine β-lyase; HCY, homocysteine; MET, methionine; MS, methionine synthase; THF, tetrahydrofolate; 5-MTHF, 5-methyltetrahydrofolate; 5,10-MTHF, 5,10-methylenetetrahydrofolate; MTHFR, methylene tetrahydrofolate reductase; SAH, S-adenosyl-homocysteine; SAHH, S-adenosyl-homocysteine hydrolase; SAM, S-adenosyl methionine; SAMS, S-adenosyl methionine synthetase; SHM, serine hydroxymethyltransferase; (**B**) Schematic representation of the changes in expression of key MTC-related enzymes and MTC metabolites in response to PVY. Black arrows show enzyme pathway and metabolite flow directions. Blue and red arrows indicate down- and up-regulation, respectively. PVY infection in Chicago plants at higher temperature causes down-regulation of key MTC (MS, SAMS, SAHH) and MTC-related folate cycle (SHM, MTHFR) enzymes, leading to the decrease of SAM and increase of SAH [5]. This would suppress SAM-dependent methylation reactions and decrease ethylene production, leading to a great increase in plant susceptibility to PVY. At normal temperature, PVY infection did not affect expression of MTC enzymes, and plants display much lower susceptibility to PVY compared with high temperature. PVY infection in Gala plants at higher temperature causes up-regulation of MS, SAMS and SHM, leading to the increase of SAM levels and SAM:SAH ratio (at 8 dpi). At higher temperature, Gala plants responded to PVY by up-regulation of CBL and SHM, also leading to increased SAM levels and SAM:SAH ratio. Gala plants remain resistant to PVY at both normal and high temperatures.

A group of proteins up-regulated at 14 dpi in PVY-infected plants growing at 22 °C included methionine synthase (MS), S-adenosylmethionine synthase 2 (SAMS), serine hydroxymethyltransferase (SHM), cytosolic ascorbate peroxidase 1, glutathione S-transferase/peroxidase and metacaspase 1 (Table S3). MS, SAMS and SHM belong to MTC (MS and SAM) or MTC-coupled folate cycle (SHM) enzymes, which are involved in MET metabolism and in plant antivirus defence [4,5] (Figure 1A). Up-regulation at 14 dpi of glutathione S-transferase/peroxidase, an antioxidant enzyme, could have an important role in ameliorating the build-up of toxic compounds. Metacaspases, as with their distant relatives, caspases, are involved in programmed cell death in plants and their up-regulation may further limit virus spread and the development of virus infection [34]. Given the general abundance of diverse proteins up-regulated under these conditions, more in-depth analysis was conducted. The g:Profiler webtool [23] was used for finding enriched GO terms in differentially regulated protein groups. According to gene ontology (GO) enrichment analysis, up-regulated proteins were mainly assigned to biological processes such as the metabolism of small molecules (GO:0044281), including organic acids (GO:0006082), oxoacids (GO:0043436) and carboxylic acid (GO:0019752). According to the Kyoto Encyclopedia of Genes and Genomes (KEGG) pathway analysis, the up-regulated proteins were enriched in pathways including metabolic pathways (KEGG:01100), carbon metabolism (KEGG:01200), amino acid metabolism (glycine, serine and threonine metabolism, KEGG:00260; alanine, aspartate and glutamate metabolism, KEGG:00250) (Table S4).

In addition, at 14 dpi in PVY-infected plants growing at 22 °C, we observed that superoxide dismutase (SOD, a biotic and abiotic stress-responsive protein, Table S3), which plays a vital role in protecting plants from reactive oxygen species (ROS)-mediated injury [35], was down-regulated in infected plants, which presumably means reduced protection against ROS damage [36] at this stage of infection. Cause–effect relationships between SOD down-regulation and PVY resistance in Gala plants remain obscure. With regards to biological processes, most of the down-regulated DE protein groups were related to oxidative stress pathways, such as cellular responses to superoxide (GO:0071451), superoxide metabolism (GO:0006801), response to superoxide (GO:0000303), cellular response to reactive oxygen species (GO:0034614), response to oxygen radical (GO:0000305), mechanisms that eliminate superoxide radicals (GO:0019430), and to translation (GO:0006412). According to the KEGG pathway analysis, down-regulated proteins were mainly involved in metabolic processes related to ribosome function (KEGG:03010), photosynthesis (KEGG:00195) and plant hormone signal transduction (KEGG:04075).

At elevated temperature (28 °C), the proteome changes were much more pronounced. In virus-infected plants at 8 dpi, we identified 291 DEP groups, 120 of which were up-regulated and 171 down-regulated (Table S3). Among the up-regulated protein groups, we identified several heat shock proteins (HSPs; Table S3) which appear to represent a defense response to elevated temperatures; this finding is consistent with our previous data, which showed that significant increases in expression of HSP genes in Gala resulted from exposure to similar elevated temperature conditions [17]. Similar to proteomic changes at 22 °C, at 28 °C we also found increased amounts of chloroplast proteins (such as photosystem II proteins and RuBisCO (ribulose bisphosphate carboxylase/oxygenase activase 1, chloroplastic)). Another remarkable finding that may relate MTC to antiviral resistance is the up-regulation of CBL (cystathionine beta-lyase) (Table S3). CBL is involved in the α,β-elimination of CYSTH (cystathionine) to form HCY (homocysteine), a precursor of MET in the MTC [36] (Figure 1A). According to GO term analysis, up-regulated proteins were mainly enriched in such biological processes as responses to hydrogen peroxide (GO:0042542), organonitrogen compound biosynthetic processes (GO:1901566), cellular amino acid metabolic processes (GO:0006520), responses to heat (GO:0009408) and responses to reactive oxygen species (GO:0000302). The KEGG pathways of up-regulated DEPs were enriched in protein processing in endoplasmic reticulum (KEGG:04141) and glycosphingolipid biosynthesis (KEGG:00603) (Table S4).

Among the most down-regulated proteins (28 °C, 8 dpi) were stress-responsive proteins such as SGRP-1 protein, superoxide dismutase (SOD), translational initiation factor eIF1 and germin (Table S3). Another group of down-regulated proteins included several histones (H2A, H2A.1, H3.2, H2B, H3.3) which may be associated with plant immune responses [37]. At these conditions (as at normal temperature), we also observed down-regulation of some ribosomal proteins (Table S3). With regards to the MTC, methionine synthase (MS) was slightly but significantly down-regulated (Table S3). GO term analysis revealed enrichment of down-regulated proteins in various processes including translation (GO:0006412), peptide biosynthetic processes (GO:0043043) and amide biosynthetic processes (GO:0043604) (Table S4). KEGG analysis showed that down-regulated proteins are mainly associated with ribosomal (KEGG:03010) and photosynthetic pathways (KEGG:00195) (Table S4).

The most pronounced proteomic response to PVY infection at elevated temperature was observed at 14 dpi. In total, 399 DE protein groups were identified including 150 up-regulated and 249 down-regulated proteins. Proteins in the up-regulated group were mainly related to stress responses and included MTC-related SHM enzyme, heat shock proteins, RuBisCO, Kunitz trypsin inhibitor, glutathione s-transferase, annexin 11 and lipoxygenase (Table S3). Interestingly, the latter was previously shown to be involved in potato defense responses against pathogens [38]. According to GO term analysis, proteins up-regulated at 14 dpi were enriched in processes such as the metabolism of small molecules (GO:0044281) including organic acids (GO:0006082), oxoacids (GO:0043436) and carboxylic acid (GO:0019752), cellular amino acid metabolic processes (GO:0006520) etc (Table S4). According to KEGG pathways analysis, protein processing in carbon metabolism (KEGG:01200) and glyoxylate and dicarboxylate metabolism (KEGG:00630) were enhanced (Table S4).

As in the case of the earlier stage of infection (8 dpi), among proteins down-regulated at 14 dpi were ribosomal proteins, SOD and SGRP-1 protein (Table S3). In addition, at 14 dpi, we observed down-regulation of transcription factor BTF3, glutamine cyclotransferase, ceramidase and some pathogenesis-related (PR) proteins such as methyl jasmonate-induced PR proteins; namely NtPRp27 and PR10 (Table S3). With regard to biological processes, the down-regulated proteins were related to photosynthesis (GO:0015979), photosynthesis, light reactions of photosynthesis (GO:0019684, GO:0009768, GO:0009765), translation (GO:0006412), peptide biosynthetic processes (GO:0043043), etc. (Table S4). According to KEGG analysis, down-regulated proteins were involved in photosynthesis (KEGG:00195), ribosome (KEGG:03010) and oxidative phosphorylation (KEGG:00196) (Table S4).

**Figure 2:**
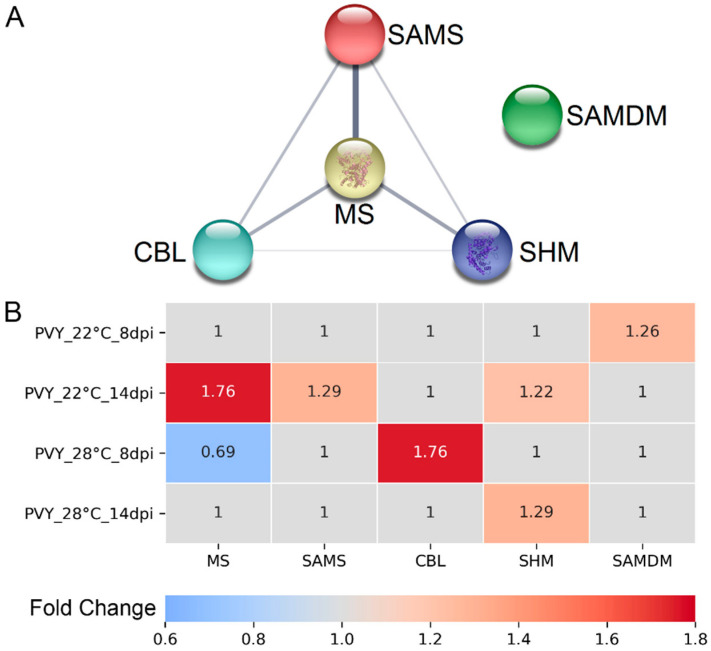
(**A**) Functional protein–protein association network of methionine cycle (MTC)-related differentially expressed proteins (DEPs) in PVY-infected Gala plants at normal and high temperature, constructed using STRING. Proteins are indicated with nodes, and links between proteins are represented by edges. CBL, cystathionine β-lyase; MS, methionine synthase; SAMS, S-adenosyl methionine synthetase; SHM, serine hydroxymethyltransferase; SAMDM, SAM dependent methyltransferase. (**B**) Heatmap showing the changes in abundance of key MTC related enzymes. PVY_22 °C _8dpi, infected Gala plants at 8 dpi at 22 °C; PVY_22 °C _14dpi, infected potato plants at 14 dpi at 22 °C; PVY_28 °C_8 dpi, infected potato plants at 8 dpi at 28 °C; PVY_28 °C _14 dpi, infected potato plants at 14 dpi at 28 °C.

We have previously shown that high temperature (28 °C) significantly increased the susceptibility of potato cultivar Chicago to PVY. This was associated with consistent changes in the expression of MTC enzymes and levels of MTC metabolites in cultivar Chicago, suggesting that the MTC plays an important role in potato responses to PVY, probably via transmethylation reactions [5]. The proteomic analysis presented above showed that cultivar Gala, resistant to PVY at both normal and elevated temperatures, also responded to PVY infection with changes in levels of key MTC or MTC-associated enzymes including MS, SAMS, CBL, SHM and SAMDM (Figure 2A). However, in contrast to coherent down-regulation of MTC enzymes in Chicago, in Gala, production of these enzymes was modulated differentially at different temperatures and at different stages of infection (Figure 2B). To examine whether the MTC-related proteomic changes are really relevant to plant responses to PVY, we selected this pathway for further analysis.

### 3.2. RNA Expression Levels of Key MTC-Related Genes

To assay whether the MTC-related proteomic changes detected above were due to transcriptional regulation, we examined how PVY infection might alter gene expression of *MS*, *SAMS*, *SAHH*, *CBL*, *SHM* and *SAMDM*, which are all key MTC or MTC-related components.

**Figure 3:**
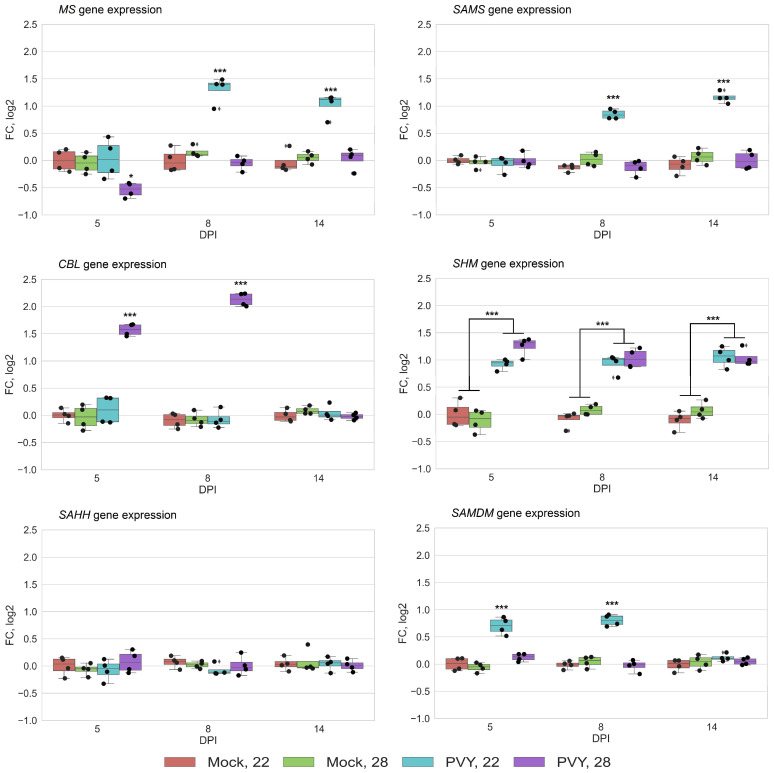
Expression level of methionine cycle (MTC)-related protein genes using quantitative reverse transcription PCR [RT-qPCR]) in systemically infected leaves of Gala plants at 22 or 28 °C at five, seven, and eight days post-inoculation (dpi), as shown. *CBL*, cystathionine β-lyase; *MS*, methionine synthase; *SAMS*, S-adenosyl methionine synthetase; *SHM*, serine hydroxymethyltransferase; *SAMDM*, SAM dependent methyltransferase. Analysis of variance and Tukey’s HSD post hoc tests were performed for RT-qPCR data. *** *p* < 0.001.

Quantitative reverse transcription PCR (RT-qPCR) analysis clearly demonstrated that systemic PVY infection at both normal (22 °C) and elevated (28 °C) temperatures induced changes in expression of *MS*, *SAMS*, *CBL* and *SHM,* but not *SAHH* (Figure 3). Moreover, these changes correlated well with rates of protein accumulation determined by proteomic analysis and strongly depended on temperature conditions and stage of infection. In particular, PVY infection at normal temperature induced persistent increases in the transcript levels of MS, SAMS and SHM from 8 dpi until the experimental endpoint (14 dpi). These transcriptional changes coincided with the increased accumulation of MS, SAMS and SHM enzymes, which was particularly evident at 14 dpi (Figure 2B and Figure 3). Interestingly, the levels of *SAMDM* gene expression also increased at the early stage of PVY infection at 22 °C (5 to 8 dpi), and this correlated well with an enhanced accumulation of the SAMDM enzyme under these conditions (Figure 3).

At elevated temperature (28 °C) we observed increases in the expression rates of *CBL* and *SHM* at early (5 to 8 dpi) and late (14 dpi) stages of PVY infection, respectively (Figure 3). These transcriptional changes were fully consistent with coincident alterations in the accumulation of CBL and SHM enzymes (Figure 2B). Meanwhile, *MS* expression was slightly reduced at 5 dpi, leading to decreased production of MS enzyme in the early stages of infection (8 dpi). Interestingly, the higher temperature (28 °C) did not affect the expression of any of these genes in mock-inoculated plants compared with 22 °C, suggesting that the individual changes in levels of their mRNA transcripts are caused by integrated responses of Gala plants to PVY infection and ambient temperature (Figure 3).

Collectively, these data imply that in contrast to Chicago plants [16], in which all key enzymes of the MTC are concertedly down-regulated in response to PVY at high temperature [16], different MTC and MTC-related enzymes in Gala plants are expressed differentially and independently of each other depending on temperature conditions and the stage of infection. These transcriptional changes could affect accumulation levels of the key MTC metabolites, which in turn could modify a wide range of transmethylation reactions and influence the outcome of virus infection.

### 3.3. Accumulation of MTC Metabolites

As SAM is the major methyl group donor in transmethylation reactions whereas SAH is a strong inhibitor of SAM-dependent methyltransferases, the ratio of SAM:SAH is commonly considered as a methylation index, whereby a decrease in this ratio causes reduced cellular methylation capacity [39]. Previously, we showed a sharp increase of PVY accumulation at elevated temperature in cv. Chicago plants correlated with decreases in SAM concentration and SAM:SAH ratios, suggesting that resistance to PVY in potato can be broken by a reduction in methylation activities [5]. Taking into account proteomic and transcriptional changes observed above in the MTC of PVY-resistant cv. Gala, we examined the effect of PVY infection on the accumulation of SAM and SAH to further elucidate possible links between methylation activities and virus resistance.

**Figure 4:**
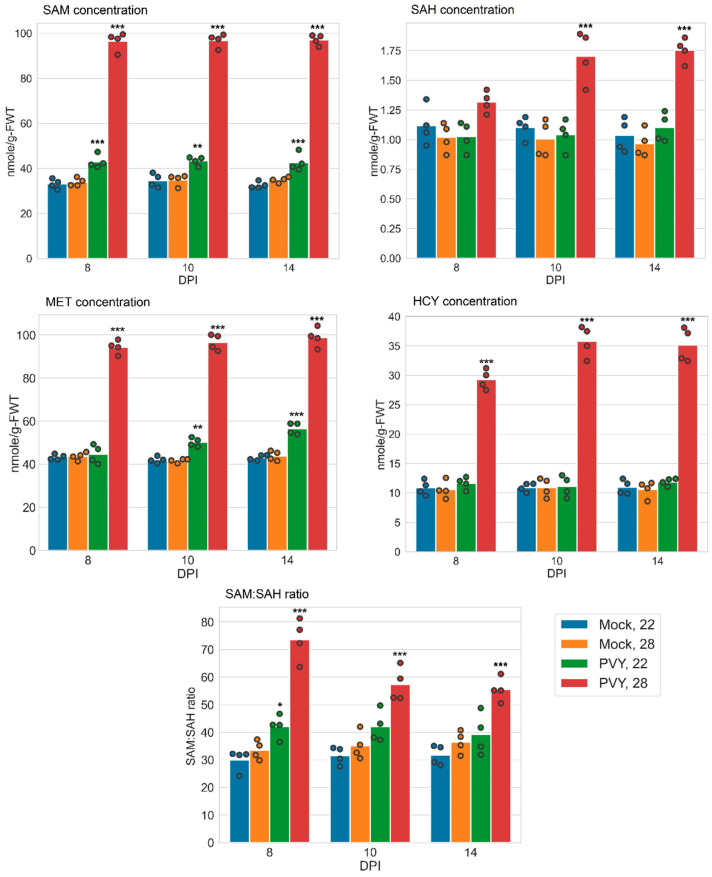
Content of methionine cycle (MTC) metabolites and SAM:SAH ratios in systemically infected leaves of potato plants at 22 or 28 °C at 8-, 10-, and 14-days post-inoculation (dpi), as shown. Content of S-adenosylmethionine (SAM), S-adenosylhomocysteine (SAH), and homocysteine (HCY) was measured by ELISA. Content of methionine (MET) was measured using a methionine assay kit (fluorometric). Analysis of variance and Tukey’s HSD post hoc tests were performed for data. * *p* < 0.05; ** *p* < 0.01; *** *p* < 0.001.

Our results indicated that PVY significantly increased SAM content at both normal (22 °C) and elevated (28 °C) temperatures compared to uninfected controls (Figure 4), although the increase at 22 °C was slighter than at 28 °C (Figure 4). The content of SAH was also increased in PVY-infected plants at 28 °C (relative to uninfected control), but not at 22 °C, and to a lesser extent than SAM (Figure 4). As a result, the SAM:SAH ratio in PVY-infected Gala plants was raised at both normal (at 8 dpi) and high temperatures relative to uninfected plants (with higher increase at 28 °C), presumably enhancing methylation processes (Figure 4) and conferring resistance to PVY.

We also compared the effects of PVY infection at elevated and normal temperatures on SAM accumulation and the SAM:SAH ratio in Gala and Chicago plants. PVY-infected Chicago plants at 28 °C had notably reduced SAM content in comparison to plants infected at 22 °C and uninfected controls [5] (Table 2). In contrast with PVY-infected Gala plants, SAM amounts were remarkably increased at both elevated and normal temperatures relative to uninfected controls and significantly exceeded those in Chicago plants (Table 2). Similarly, SAM:SAH ratios in PVY-infected Gala plants were higher than in Chicago at both temperatures with the most pronounced difference observed at elevated temperature; SAM:SAH was dramatically reduced in Chicago but increased in Gala, particularly at the higher temperature (Table 2).

Collectively, these data clearly indicate that Gala and Chicago respond to PVY differentially, modulating the accumulation of SAM and SAM:SAH ratio in opposite directions. PVY-resistance in Gala plants is associated with the increase in SAM content and SAM:SAH ratio, whereas sharp rises in susceptibility to PVY in Chicago plants at high temperature is accompanied by their decrease. This suggests that modulation of methylation capacity regulated by SAM:SAH plays an important role in determining plant resistance or susceptibility in PVY–plant interactions.

In PVY-infected Gala plants at normal temperature, the elevated transcriptional levels of *MS* and *SHM* could facilitate the synthesis of MET, which should concomitantly increase the content of SAM. This prediction was confirmed by measuring levels of MET (Figure 4). Increases in expression of *SAMS* possibly additionally contributes to overproduction of SAM (Figure 4). There are also other alternate metabolic routes in PVY-infected Gala plants grown at higher temperatures, which can lead to the overproduction of SAM. For example, enhanced transcription of *CBL* (in spite of some reduction in *MS* transcription) leads to an increase in HCY content (Figure 4), which is a precursor of MET and SAM.

## 4. Discussion

Viruses are major plant pathogens that cause approximately half of all emerging plant disease outbreaks [40]. Of significant concern for potato cultivation are potyviruses, such as PVY. Losses of up to 45% of production have been estimated following primary PVY infection, but the largest losses are experienced when the crop is grown from PVY-infected seed (secondary infection), where yield reductions of up to 64% have been reported [41,42] depending on environmental conditions. One of the key environmental factors affecting plant-PVY interactions is temperature, which, according to climate change scenarios [15], is likely to be increased during the potato growing season. Many but not all defence responses to PVY can break down at high temperatures [43].

In previous work, we showed that potato cv. Gala exhibits a distinct type of PVY resistance that is manifested as very low but detectable levels of virus accumulation in both inoculated and non-inoculated leaves; moreover, these levels were not significantly affected by elevated temperature [16]. In cv. Chicago, the pattern of PVY accumulation in inoculated leaves was essentially similar to that in Gala. However, in non-inoculated leaves, great differences in the rates of PVY accumulation were observed between Chicago and Gala. In contrast to Gala plants, some significant rise in PVY levels was detected in the upper leaves at normal (22 °C) temperature (up to seven-fold) and was further dramatically increased (a 30-fold increase) at elevated (28 °C) temperature [16]. In more recent work, we demonstrated that the enhanced susceptibility of Chicago plants to PVY at 28 °C may be orchestrated by downregulation of MTC enzymes, which disturbs normal functioning of the cycle and suggests an important role of MTC in modulating defense responses to PVY [5].

To provide new insights into the mechanisms of PVY resistance operating in Gala, here, we used iTRAQ-based proteomic technology, which was also utilized in an earlier study to examine the responses of Chicago plants to PVY. Overall, the proteomic responses of the resistant cultivar Gala to PVY infection were markedly stronger than those of cv. Chicago. At normal temperature (22 °C) in PVY-infected Gala plants, we identified 43 DEP groups, including 11 up-regulated and 32 down-regulated proteins at 8 dpi, and 189 groups (106 up-regulated and 83 down-regulated) at 14 dpi compared with mock-inoculated plants. In contrast, Chicago plants treated under the same conditions gave only 16 and 23 DEPs at 8 and 14 dpi, respectively [5]. Among the up-regulated DEPs in Gala that could potentially contribute to PVY resistance were a WRKY transcription factor, which is involved in regulation of various biological processes (including stress responses); various stress-responsive proteins; chloroplast proteins (some of which are key components of early immune responses); HSPs, which are also known to facilitate virus resistance [44]; and several MTC-associated enzymes (namely MS, SAMS, SHM). Interestingly, neither of these proteins were up-regulated during PVY infection of Chicago. Down-regulated Gala DEPs include proteins involved in photosynthesis and ribosomal proteins.

In addition to the up-regulation of HSPs and chloroplast proteins found in PVY-infected Gala plants at normal temperature, proteomic changes in these plants at higher temperature also included the up-regulation of MTC-associated CBL and SHM. Among down-regulated DEPs were PR, photosynthetic and ribosomal proteins. Interestingly, chloroplast proteins that were up-regulated in Gala were down-regulated in Chicago, whereas PR-proteins were down-regulated in Gala but up-regulated in Chicago.

Although proteomic studies highlight the potential functions of some of the identified host DEPs during viral infections, additional experiments are required to elucidate the molecular mechanisms of plant–virus interactions underpinning the host resistance. Earlier, we found that plant responses to PVY in Chicago are closely related to the MTC [5] and showed that the increased susceptibility of Chicago plants to PVY at higher temperature was associated with the orchestrated down-regulation of levels of major enzymes involved in the MTC (MS, SAMS, SAHH) and MTC-related folate cycle (SHM and MTHFR) at 28 °C. These changes were consistent with the expression rates of the corresponding genes and led to reduction of HCY and MET as sequential precursors of SAM. This consequently led to a decrease in the levels of SAM itself as a methyl donor of SAM-dependent methyltransferases. At the same time, decreases in SAHH levels led to an increase in SAH accumulation, which is a substrate for SAHH (Figure 1B). These changes significantly decreased the value of SAM:SAH ratio, which is usually regarded as an indicator of transmethylation activities [4,39], suggesting functional interplay between the high susceptibility to PVY in Chicago at elevated temperature, disturbance of the MTC, and transmethylation. This was further supported by the ability of exogeneous MET to restore the accumulation of MTC metabolites (including SAM:SAH ratio) and subvert the susceptibility to PVY at these conditions [5]. Interestingly, at 22 °C, changes in the expression of MTC-related enzymes in PVY-infected Chicago were not observed compared with mock-inoculated plants, and susceptibility to PVY was much lower than that at 28 °C [5].

In the resistant cultivar Gala, the pattern of MTC changes in response to PVY was quite different from that in Chicago plants. In contrast to Chicago, MTC-related enzymes and their corresponding genes were not downregulated, but some of them were upregulated. Moreover, different sets of MTC-related enzymes were upregulated in this cultivar depending on (22 °C or 28 °C) temperature (Figure 1B). However, in spite of different sets of MTC-related enzymes being up-regulated at 22 °C (MS, SAMS, SHM), or 28 °C (CBL and SHM) (Figure 1B), significant increases in the accumulation of MET and SAM were observed at both temperatures. As a result of these changes, the SAM:SAH ratio was also increased at both temperatures (Figure 4 and Table 2), with the most striking increase observed at 28 °C. Interestingly, under these conditions, the SAM:SAH ratio in Chicago was dramatically reduced, leading to a great difference between Gala (73.61 ± 3.78) and Chicago (3.62 ± 0.23) (Table 2).

At 22 °C, SAM accumulation as well as the SAM:SAH ratio were also significantly higher (although to lesser extent) in PVY-infected Gala than in Chicago plants (42.99 ± 1.49 versus 28.02 ± 0.34 for SAM; and 42.16 ± 2.10 versus 30.98 ± 0.61 for SAM:SAH) (Table 2). Thus, the data presented here show clear relationships between PVY resistance and the accumulation of SAM and SAM:SAH ratio, suggesting a positive regulatory role of the MTC and transmethylation in conferring virus resistance in potato. One possibility is that the antivirus RNAi-based defence mechanism may be strengthened by the stabilisation of virus-specific siRNAs via methylation processes, which use the SAM-dependent HEN1 methyltransferase. Another possible mechanism may be related to the epigenetic methylation of host DNA or histones, which is also known to be involved in responses to biotic and abiotic stresses [45,46]. An additional factor released as a product of the MTC-related pathway is ethylene, a plant hormone that, as with other hormones, plays important roles in plant responses to viruses [14]. An increased accumulation of SAM, which is a key component in the synthesis of ethylene, may lead to increases in ethylene levels and consequently enhanced virus resistance.

It has also been shown that resistance to PVY in potato may be associated with other plant defence responses such as the salicylic acid-dependent resistance pathway [17,28]. Thus, it seems unlikely that controlling the resistance or susceptibility of potato plants to PVY relies on a single regulatory mechanism [16,28]. Moreover, elevated temperatures can modulate various plant responses to virus infections differentially in different cultivars, as exemplified in this work on MTC-based responses. These data conform to our previously published report [16], suggesting that responses to PVY infection and stress caused by elevated temperatures in potato have some common underlying mechanisms that may be integrated in specific consolidated networks controlling plant sensitivity to the virus in a temperature-dependent and cultivar-specific manner. Therefore, further elucidation of such regulatory networks is required for effective control of virus diseases in the face of climate change.

## 5. Conclusions

We have previously shown that the main enzymes of MTC were down-regulated in PVY-infected Chicago plants at higher temperatures, causing a consistent and concerted reduction in the levels of the major MTC metabolites including the universal methyl group donor SAM. This suggested that the enhanced susceptibility of Chicago potato plants to PVY at high temperatures may be triggered by the MTC perturbation and concomitant reduction in transmethylation activities [5]. Remarkably, as in the case of Chicago, here, we revealed significant changes in the expression of MTC and MTC-associated enzymes induced by PVY infection in cv. Gala. However, in contrast to Chicago, with Gala plants, the expression of a different set of enzymes was affected and this ultimately led to an increase in the levels of SAM and its immediate precursors—MET and HCY. Thus, we suggest that resistance to PVY in potato cultivars may be positively regulated by intracellular amounts of MTC metabolites, which determines the rate of methylation.

## Figures and Tables

**Table 1:** Numbers of peptides and corresponding protein groups identified by liquid chromatography with mass spectrometry (LC–MS/MS).

Identifications	PVY 22 °C 8 dpi	PVY 28 °C 8 dpi	PVY 22 °C 14 dpi	PVY 28 °C 14 dpi
Peptides	11,353	14,106	12,893	13,703
Protein groups	2386	2693	2722	2619

**Table 2:** SAM levels and SAM:SAH ratio in PVY-infected Gala and Chicago plants at 8 dpi.

Samples	SAM (nmol/g-FWT)		SAM:SAH	
	Gala	Chicago	Gala	Chicago
Mock 22 °C	33.15 ± 1.07	27.94 ± 0.36	30.13 ± 1.98	33.32 ± 0.63
Mock 28 °C	33.95 ± 0.89	27.16 ± 0.61	33.56 ± 1.70	29.78 ± 0.82
PVY 22 °C	42.99 ± 1.49 **	28.02 ± 0.34	42.16 ± 2.10 *	30.98 ± 0.61
PVY 28 °C	96.349 ± 2.05 ***	10.96 ± 0.51	73.61 ± 3.78 ***	3.62 ± 0.23

Contents of S-adenosylmethionine (SAM) and S-adenosylhomocysteine (SAH) were measured in Gala and Chicago [5] plants by ELISA. Differences in SAM levels and SAM:SAH ratio between PVY-infected Gala and Chicago plants at both 22 °C and 28 °C are significant according to the Tukey’s HSD post hoc test: * *p* < 0.05; ** *p* < 0.01; *** *p* < 0.001.

## Data Availability

The mass spectrometry proteomics data have been deposited to the ProteomeXchange Consortium at http://www.proteomexchange.org/via (accessed on 20 May 2021) the PRIDE partner repository with the data set identifier PXD020495. The other data that support the findings of this study are available from the corresponding author upon reasonable request.

## Supplementary Materials

The following are available online at https://www.mdpi.com/article/10.3390/v13060955/s1, Table S1: Primers used for quantitative RT PCR, Table S2: The list of identified proteins, Table S3: The list of differentially regulated proteins, Table S4: The results of functional enrichment analysis by g:Profiler (https://biit.cs.ut.ee/gprofiler; accessed on 12 April 2021).
