# Supplementary material for: The Resistance Responses of Potato Plants to Potato Virus Y Are Associated with an Increased Cellular Methionine Content and an Altered SAM:SAH Methylation Index
Source: Viruses. 2021 May 21;13(6):955. doi: 10.3390/v13060955 (PMC8224460; doi:10.3390/v13060955)
Supplement: Supplementary file 1 [file viruses-13-00955-s001.zip › TableS1_Gala.pdf]

Table 1. Primers used for quantitative RT PCR

Primer	5′-3′ sequence	Reference	Primer Concentration (nM)	E (%)
PVY^O-F<br>PVY^O-R	TATGATGGATTTGGCGACCACTTGT<br>TAAACTAGGCAGCTCTGCATCATG	Makarova et al., 2018	400	95.7
StMS-F<br>StMS-R	GTTAGTGAATACAAGGAGGCT<br>GCTGCTTTTGATAACAAGAGG	PGSC0003DMG400008708*	400	98.5
StSAMS-F<br>StSAMSR	ATCACGACCAAGGCTATTGT<br>CAGGACTTTGCTGCTCAATG	PGSC0003DMG400005855*	400	94.5
StSAHH-F<br>StSAHH-R	AACTTTGCTTTTCCCTGCT<br>CGGCAATAAGGGCAACC	PGSC0003DMG400004572*	350	94.5
StSHM-F<br>StSHM-R	AGGTTGCTGATAAATGTGGG<br>CTCTTGTGTGTGGTGGTAG	PGSC0003DMG400015745*	350	93. 4
StCBL-F<br>StCBL-R	TCTGCCTACCAACACAACC<br>TCCATCTCTTTGTCTCTTGAGC	PGSC0003DMG400029836*	350	96.2
StSAMDM-F<br>StSAMDM-R	TGATGGAAAGGTTTTGCCAC<br>AATTCACCATGAGACGACCA	PGSC0003DMG400005855*	400	97.5
StCOX-F<br>StCOX-R	GGTCGGACATACCTGAAAC<br>CCAAAAGTATGAAAAGCTGGAG	Baebeler et al., 2011	350	97.3
StEF-1α-F<br>StEF-1α-R	CTTGACGCTCTTGACCAGATT<br>GAAGACGGAGGGGTTTGTCT	Nicot et al., 2005	350	98.7

Full references are provided in the main text. Primer concentrations giving the lowest threshold cycle (C_t) value were utilized in RT-PCR and are listed in the Table. E, efficiency of PCR amplification as calculated by CFX Manager Software. *Gene sequences were retrieved from Plant Genomics Resource Phytozome 12 (https://phytozome.jgi.doe.gov/pz/portal.html).
